# Cell Analysis from Dried Blood Spots: New Opportunities in Immunology, Hematology, and Infectious Diseases

**DOI:** 10.1002/advs.202100323

**Published:** 2021-07-18

**Authors:** Ines Ait Belkacem, Noushine Mossadegh‐keller, Penelope Bourgoin, Isabelle Arnoux, Marie Loosveld, Pierre‐emmanuel Morange, Thibaut Markarian, Pierre Michelet, Jean Marc Busnel, Sandrine Roulland, Franck Galland, Fabrice Malergue

**Affiliations:** ^1^ Department of Research and Development Beckman Coulter Life Sciences‐Immunotech 130 Avenue de Lattre de Tassigny Marseille 13009 France; ^2^ Aix Marseille Université CNRS INSERM CIML Centre d'Immunologie de Marseille‐Luminy Marseille 13009 France; ^3^ Department of Hematology Laboratory Timone University Hospital APHM 264 Rue Saint‐Pierre Marseille 13005 France; ^4^ Aix Marseille Université INSERM INRAE C2VN, 27 Boulevard Jean Moulin Marseille 13385 France; ^5^ Department of Emergency Medicine and Intensive Care Timone University Hospital APHM 264 Rue Saint Pierre Marseille 13005 France

**Keywords:** analysis, cells, dried blood spots

## Abstract

Blood cell analysis is a major pillar of biomedical research and healthcare. These analyses are performed in central laboratories. Rapid shipment from collection site to the central laboratories is currently needed because cells and biomarkers degrade rapidly. The dried blood spot from a fingerstick allows the preservation of cellular molecules for months but entire cells are never recovered. Here leucocyte elution is optimized from dried blood spots. Flow cytometry and mRNA expression profiling are used to analyze the recovered cells. 50–70% of the leucocytes that are dried on a polyester solid support via elution after shaking the support with buffer are recovered. While red blood cells lyse upon drying, it is found that the majority of leucocytes are preserved. Leucocytes have an altered structure that is improved by adding fixative in the elution buffer. Leucocytes are permeabilized, allowing an easy staining of all cellular compartments. Common immunophenotyping and mRNAs are preserved. The ability of a new biomarker (CD169) to discriminate between patients with and without Severe Acute Respiratory Syndrome induced by Coronavirus 2 (SARS‐CoV‐2) infections is also preserved. Leucocytes from blood can be dried, shipped, and/or stored for at least 1 month, then recovered for a wide variety of analyses, potentially facilitating biomedical applications worldwide.

## Graphical abstract

A new method is described in which blood is dried on a polyester solid support, shipped, and/or stored at room temperature for at least 1 month. Leucocytes are recovered for a wide variety of biomedical analyses. The method has significant implications for resource limited settings and may facilitate patient management worldwide.

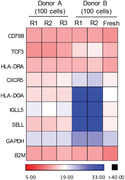

## 1 Introduction

The analysis of blood‐derived cells plays a central role in biomedical research and healthcare. Cells of interest are mostly blood leucocytes, bone marrow aspirates, cord blood stem cells, tissue extracts, or cultured cells. The analysis is generally performed by specialized operators in dedicated central laboratories with high‐technology equipment and reagents. However, the sources of cells—patients, participants in multicentric studies, or animals—are not always near those laboratories. This means that cell samples must be shipped or that patients need to travel. Especially during periods of quarantine, it would be convenient to enable self‐collection and shipment of blood samples to the laboratory by courier.

Cells are fragile and degrade rapidly in sampling tubes when stored for hours or days. This major challenge has led to the development of multiple methods to preserve cells for a few days or even up to many years. Preservation of viable mammalian cells, however, requires high concentrations of cryoprotectants such as dimethyl sulfoxide or glycerol, and extremely low temperatures (−80 °C freezers or −196 °C liquid nitrogen for storage and dry ice for shipping).^[^ ^1^ ^]^ Although cryogenic temperatures are now widely used for long‐term storage of a wide range of cell lines, tissues, and peripheral blood mononuclear cells (PBMCs), it is costly and time consuming to prepare, store, transport and recover these frozen samples. Preparing PMBCs further induces a significant loss of white blood cells, including loss of neutrophils and eosinophils (granulocytes), and, moreover, requires large volumes of blood.

Other methods of whole blood preservation at room temperature (RT), or at 4 °C, have been developed, based on the addition of stabilizers such as protease inhibitors, DNase, RNase, and fixatives. Blood can be preserved in those conditions for several days. This method does not require washing and therefore does not lead to significant cell loss, but some markers are affected, and cell viability is lost.^[^ ^2^ ^]^ In addition, the dedicated tubes, prefilled with reagents, are costly, and shipping, although simpler, still requires dedicated solutions. Also, these tubes need to be filled with blood even for a single analysis as they contain a fixed amount of preservative intended for a full tube of blood.

Bang introduced the dried blood spot (DBS) sampling method in 1913 for glycemia.^[^ ^3^ ^]^ Guthrie generalized the method in 1963 for neonatal screening for phenylketonuria.^[^ ^4^ ^]^ DBS is now the reference sampling method for the analysis of biochemical markers and nucleic acids. It allows screening of various diseases, using a heel‐prick.^[^ ^5^, ^6^, ^7^ ^]^ Beyond newborns, DBS have also been proposed in therapeutic drug monitoring, infectious disease diagnosis, metabolomics and proteomics.^[^ ^8^, ^9^, ^10^ ^]^ DBS provides a number of advantages: Storage requires little space and does not need low temperatures, even for prolonged storage. Shipping is much easier, cheaper, and safer than frozen and stabilized samples. DBS cannot leak or be broken in transit. Furthermore, there is no requirement for carriage on dry ice.^[^ ^11^ ^]^ Also, DBS represents a low infection hazard as some viruses are inactivated due to disruption of their envelopes upon drying.^[^ ^12^, ^13^ ^]^

To our knowledge, there is no publication describing preservation and analysis of entire leucocytes recovered from DBS. Inspired by the simplicity of the fingerstick and DBS method, we aimed to develop a new method for drying whole blood on a solid support, as well as a simple and gentle recovery procedure compatible with cell analysis. As a proof of concept, we studied the preservation of leucocyte subpopulations, their variety of surface and intracellular proteins, and their nucleic acid contents after cell sorting. Considering the difficulties to implement large scale diagnosis, illustrated by the current Coronavirus disease of 2019 (COVID‐19) pandemic, we further evaluated the preservation of the CD169 marker on a cohort of COVID‐19 patients, since CD169 was recently described by our team and others as a relevant biomarker of acute viral infections such as Severe Acute Respiratory Syndrome induced by Coronavirus 2 (SARS‐CoV‐2).^[^ ^14^, ^15^, ^16^ ^]^

## 2 Results

### 2.1 Recovering Leucocytes from a Dried Blood Spot: Preliminary Testing

**Figure 1:**
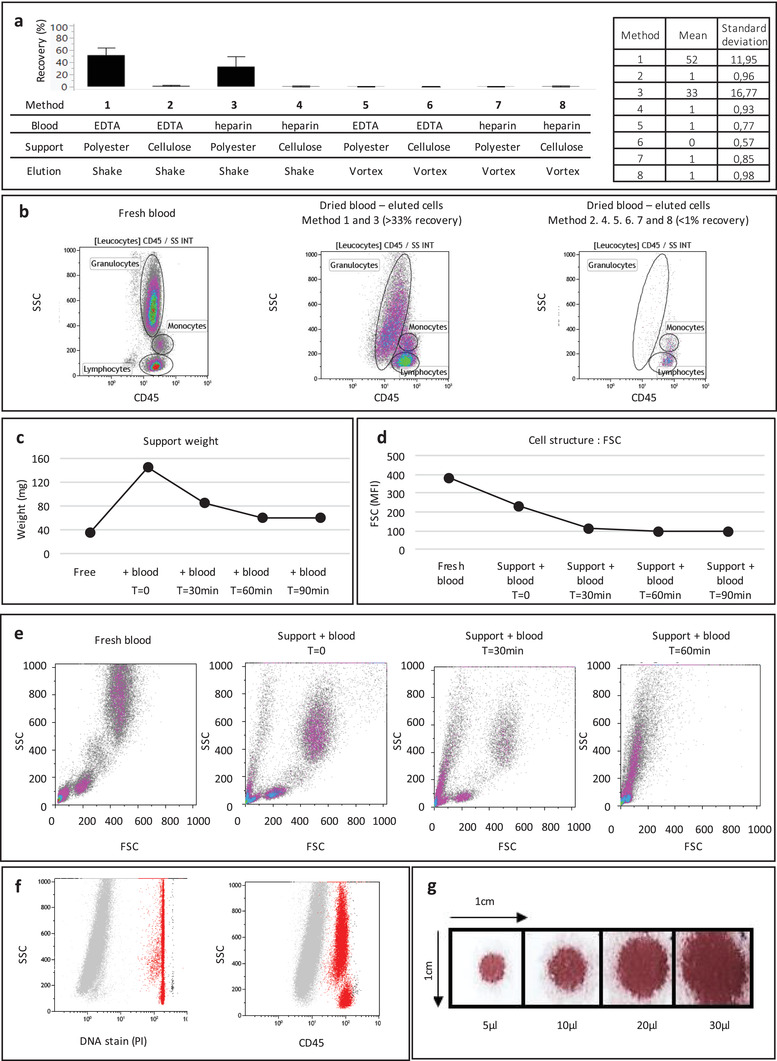
Recovering leucocytes from a DBS stored 24 h at RT. a) Leucocyte recovery quantification on six donors in each method, data are presented as mean ± standard deviation. b) Representative flow cytometry dot plots and leucocyte subpopulation frequencies. c) Solid support weight before and after blood spotting at *t* = 0, 30, 60, 90 min. d) Quantification of leucocyte FSC from fresh blood and dried blood at: *t* = 0, 30, 60 min and e) representative flow cytometry plots. f) PI and CD45 staining of recovered leucocytes. g) DBS size with increasing blood volumes.

Flow cytometry experiments showed that DBS cellulose solid support did not allow recovery of more than 1% of cells (Methods 2, 4, 6, and 8). The polyester solid support yielded a recovery of 33% with heparinized blood (Method 3) or 52% with ethylenediaminetetraacetic acid (EDTA) blood (Method 1). Furthermore, manual shaking (Methods 1–4) allowed a higher recovery than vortexing (Methods 5–8) (**Figure** **1**a). Principal leucocyte subpopulation (lymphocytes, monocytes, and granulocytes) were preserved, but granularity (SSC) was impaired in granulocytes (Figure 1b).

Weighing the solid support every 30 min after blood spotting showed no further weight change after 60 min at RT (18–25 °C) and local levels of humidity (30–60%) (Figure 1c). Cell size and granularity were strongly decreased, showing that leucocyte structural properties were impaired during the drying and not completely restored upon elution (Figure 1d,e). Leucocyte staining with a nonpermeant DNA probe and a pan‐leucocyte marker showed that all eluted cells were permeabilized (Figure 1f).

We determined that 20 µL of blood could be spotted onto a 1 cm² solid support and estimated that it is possible to recover 50 000 to 100 000 cells from that 20 µL sample, assuming 50% recovery and normal leucocyte count (5000 to 10 000 µL^−1^) (Figure 1g).

### 2.2 Recovering Leucocytes from a Dried Blood Spot: Method Optimization

Recovery was not impaired when we replaced red blood cells (RBC) lysing solution with phosphate‐buffered saline 1X (PBS 1X). This suggests that RBCs are already lysed by the drying (Figure S1, Supporting Information). Adding a fixative (formaldehyde, FA) to the elution buffer at low concentration (0.025 and 0.05%) improved structure integrity without impairing leucocyte CD45 staining. Formaldehyde 0.05% also improved the recovery of neutrophils (the most fragile leucocytes) and thereby restored subpopulation frequencies to pre‐DBS levels. When the concentration of formaldehyde was 0.1% or higher, staining was impaired.

We compared different elution methods (vortex, rotating mixer, automated orbital shaker and manual shaking). The polyester solid support layers appeared to disintegrate when tubes were manually shaken up by 15 up‐and‐down movements. This corresponded to the best leucocyte recovery (Figure S2, Supporting Information).

Since EDTA‐anticoagulated blood allowed better leucocyte recovery than heparin‐anticoagulated blood (52% and 33% respectively) (Figure 1a), we evaluated the role of these compounds on the system. The solid support was therefore pretreated with heparin or EDTA. Heparin induced a decrease in leucocyte recovery of EDTA‐anticoagulated blood (from 55% to 8%) whereas EDTA pretreatments improved leucocyte recovery of both EDTA‐ (from 55% to 77%) and heparin‐anticoagulated blood (from 36% to 54%) (Figure S3a, Supporting Information). This adverse effect could be linked to the large polyanionic structure of the heparin. In line with this hypothesis, pretreating the solid support with a large polycationic molecule (poly‐l‐lysine) improved leucocyte recovery on heparinized blood (from 13% to 40%) and had no effect on EDTA‐anticoagulated blood (Figure S3b, Supporting Information). Since proteases could be released from permeabilized leucocytes and impact their structure and markers and thereby the recovery, we pretreated the solid support with protease inhibitors. This treatment improved the recovery of both EDTA‐anticoagulated blood (from 47% to 76%) and heparin‐anticoagulated blood (from 36% to 68%) (Figure S3c, Supporting Information).

When testing different storage conditions (from 4 to 37 °C), similar leucocyte recoveries and subpopulation frequencies were obtained. The only change observed was a decrease in the granularity (SSC) (Figure S4, Supporting Information).

We concluded that the simplest method allowed a satisfactory recovery (about 50%): EDTA‐anticoagulated blood is dried and stored at RT, using an untreated polyester solid support, and later eluted with PBS 1 × 0.05% FA and manual shaking. We therefore selected this method for the following experiments. If necessary, better recovery may be achieved by pretreating the solid support.

### 2.3 Cell Marker Preservation Analysis

**Figure 2:**
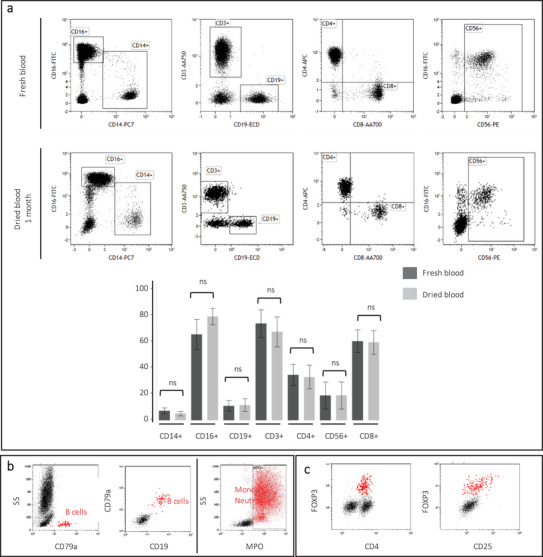
Marker preservation in DBS. a) General surface phenotyping markers: CD14, CD16, CD19, CD3, CD4, CD56, and CD8 after 1 month storage at RT. Comparison between fresh and dried blood on Student's *T*‐test, ns: nonsignificant (*p*‐values > 0,05); *: significant (*p* values ≤ 0,05); *n* = 10 donors per condition, data are presented as mean ± standard deviation. b) Intracellular leukemia orientation markers: CD79a and MPO and c) intracellular Tregs marker: FoxP3 after 1 month storage at RT.

We performed multicolor flow cytometry analysis to evaluate the preservation of multiple leucocyte subpopulations based on their relevant surface markers. CD3, CD4, CD8, CD14, CD16, CD19, CD45, and CD56 and the corresponding subset proportions were all preserved after 1 month of storage (**Figure** **2**a and Table S1, Supporting Information). The background increased for all markers, reducing the signal‐to‐noise ratio.

Since the cells were permeabilized, we also evaluated the preservation and accessibility of common intracellular markers. The myeloid granule marker (myeloperoxidase, MPO) and the B cell cytoplasmic marker CD79a, both widely used to classify acute myeloid and lymphoid leukemia, and the regulatory T cells (Tregs) nuclear marker FoxP3, broadly studied in the context of autoimmunity, were easily detected after 1 month of storage (Figure 2b,c).

### 2.4 Venous versus Capillary Dried Blood Comparison

As we used venous blood samples anticoagulated with EDTA for the preliminary and optimization experiments, we compared whether blood drawn from a lancet fingerstick directly onto the solid support can yield similar cell recovery and marker preservation, and if the solid support needs to be pretreated with anticoagulant. We compared the results from three donors and showed that all common markers and the corresponding subset proportions were preserved in all the conditions. Recovery was not significantly different between anticoagulated venous and nonanticoagulated capillary dried blood. Solid support pretreatment with EDTA is not necessary (Figure S5, Supporting Information).

### 2.5 Preservation of mRNA Integrity after Cell Sorting

**Figure 3:**
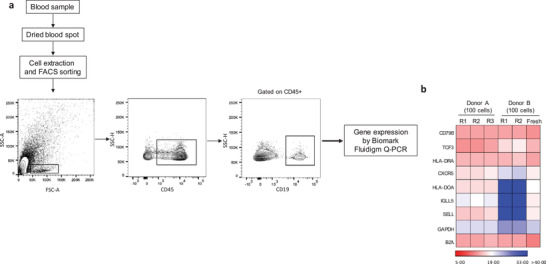
Gene expression profiling of B cells from DBS stored 24 h at RT. a) Representative FACS profiles of the sorting strategy from DBS CD45+CD19+ B cell populations. b) Gene expression analysis of CD19+ B cells isolated from two donors using DBS (R1, R2, R3 from donor A; R1, R2 from donor B) and fresh blood (from donor B). Expression levels (expressed as Ct values) of B cell‐related genes were determined using nanofluidic Fluidigm array real‐time PCR. *R* represents the number of replicates per donor.

To evaluate whether DBS storage maintains the integrity of mRNA molecules in leucocytes, we analyzed the expression profiles of seven B cell related genes (*CD79B, TCF3, HLA‐DR, CXCR5, HLA‐DOA, IGLL5*, and *SELL*) and two housekeeping genes (*B2M and GAPDH*) on CD45^+^CD19^+^ B cells (100 cells per replicate) sorted from DBS from two healthy donors and compared to fresh‐sorted B cells from the same donor (**Figure** **3** and Figure S6 and Table S2, Supporting Information).We found that housekeeping genes *B2M* and to a lesser extent *GAPDH* are expressed in all DBS replicates as well as in fresh blood, confirming the ability to measure mRNA expression after DBS processing. We further confirmed the expression of typical B‐cell markers *CD79b* and *CXCR5* that are expressed on all circulating mature B cells. The detection of certain genes (such as *HLA‐DOA* or *SELL*) is heterogeneous depending on the blood donors. This might be influenced by the proportion of circulating naive versus memory B cell subpopulations in a given individual. The overall results indicate that the B cell expression signature is preserved from our modified DBS procedure.

### 2.6 Preservation of CD169 from COVID‐19+ Patient Samples

**Figure 4:**
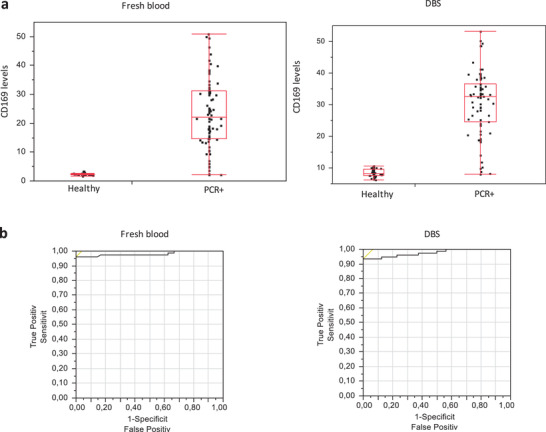
Preservation of CD169 from COVID‐19+ patient samples on DBS stored one week at RT; *n* = 76. a) CD169 Index in fresh and dried blood and b) corresponding ROC curve.

In order to validate the use of the DBS strategy in a clinical diagnosis, we took advantage of the ability of the CD169 marker to discriminate SARS‐CoV‐2 infections and compared 76 patients with positive SARS‐CoV‐2 real time polymerase chain reaction (RT‐PCR) to 48 healthy volunteers (**Figure** **4**). In total, 39 (51%) women and 37 (49%) men, with a mean age of 60 ± 18 years and SARS‐CoV‐2 mean RT‐PCR level of 24.2 ± 5.8 Cycle Threshold (CT) were included. Using flow cytometry, we observed that in the control method (fresh blood) as well as in the DBS method, patients with SARS‐CoV‐2 infections had significantly higher CD169 levels (23.37 ± 11.99 and 30.83 ± 10.32 respectively) than healthy volunteers (2.26 ± 0.32 and 8.46 ± 1.28 respectively). Receiver operating characteristic (ROC) analysis showed area under the curve (AUC) of 0.981 and 0.976, respectively. Sensitivity (96%) and specificity (100%) were obtained in the control method. Similar sensitivity (93%) and specificity (100%) were calculated for the DBS method, using the optimal cutoff values (greater than or equal to 3.55 on fresh blood, and 11.73 on DBS). Again, we noticed an increase in the background and staining levels in the DBS.

## 3 Discussion

In this study, we developed a new method for preserving blood on a solid support for cell analysis. After multiple optimizations, we were able to recover and analyze leucocytes from a DBS. Recoveries ranging from 50% to 70% were achieved, which is equivalent to or even better than PBMC cryopreservation, with the added advantage of preserving granulocytes.^[^ ^17^ ^]^ A few microliters of blood are sufficient for analysis and can be stored for at least 1 month at room temperature. This sample size provides the recovery of thousands of cells. Such quantities are suitable for numerous applications. Recovered cells can be analyzed with various probes and antibodies to easily detect not only surface but also intracellular markers. Both flow cytometry‐based analysis and sorting are made possible by the proposed strategy. Subsequent mRNA quantification on DBS‐collected cells was also successful.

Different types of DBS solid supports are available on the market. They are almost all made of pure cotton (cellulose) and can vary in pore size or thickness.^[^ ^18^ ^]^ They can be untreated or modified with various chemical solutions in order to increase stability of analytes or in any other way to improve the performance of a DBS sample.^[^ ^19^, ^20^ ^]^ In this study, we found that leucocyte recovery was not possible from cellulose solid support, whereas we achieved a good recovery with a polyester solid support. This may be due to the structural properties of each solid support. Cellulose solid support is a network of thin fibers with tight mesh that form pores about .0.2–1,2 µm diameter, which may not allow leucocyte (5–20 µm) holding, whereas polyester solid support is made of thicker fibers forming pores about 0.5–50 µm diameter. Also, the larger surface area of the cellulose may cause more and tighter cell adhesion. Furthermore, the plasma gas treatment of the polyester solid support by the manufacturer is described as improving the biointeraction with leucocytes.

Eluting leucocytes with a strong manual shaking provided the best recovery. This could be due to the lateral movement causes the solid support layers to break apart and to resuspend the cells. This step could be easily automated in the future.

Principal subpopulation proportions (lymphocytes, monocytes and granulocytes) were preserved. RBCs were automatically lysed and the recovered leucocytes were all permeabilized. This provided a path toward easy intracellular staining without the need for a separate permeabilization step. Recovered leucocytes had an impaired structure (reduced cell size and granularity), but adding a low fixative concentration (0.05% formaldehyde) improved their structural integrity without impairing antigen staining. In view of recent alerts on the carcinogenic risk of FA, this approach uses much less FA than current flow cytometry techniques and, therefore, may have health, ecological and sustainability impacts on top of being very straightforward.

Blood drying and storage were achieved at room temperature and humidity levels. Of course, these conditions may vary but can be easily controlled. DBS storage at a higher temperature (37°) slightly impaired the cell structure but provided similar leucocyte recovery and subpopulation frequencies. DBS are usually stored at RT and all our experiments worked at RT. Still, some analytes have been described as being sensitive to temperature (e.g., human immunodeficiency virus (HIV) RNA and C‐reactive protein). In these cases, stability may be improved by storing the sample at lower temperatures).^[^ ^21^, ^22^, ^23^, ^24^ ^]^ Humidity is reported to affect sample quality by causing bacterial growth, degrading molecules of interest, or inducing a variation in extraction recovery.^[^ ^11^ ^]^ Complete drying is therefore preferable and can be easily achieved anywhere with desiccant added to an impermeable packaging.^[^ ^5^ ^]^

Stable molecules are well preserved by drying, but the degradation or modification of less stable molecules can present a problem.^[^ ^18^, ^23^, ^25^ ^]^ Multiple treatments of the solid support have been described to preserve specific analytes with traditional DBS.^[^ ^26^ ^]^ In our hands, EDTA, poly‐l‐lysine, or protease inhibitor pretreatment improved the recovery slightly.

Using flow cytometry, we evaluated surface markers that monitor immune status, intracellular markers that classify hematological disorders, and an intranuclear marker that is relevant to autoimmune conditions. Although the background increased for all markers, reducing the signal‐to‐noise ratio, all markers tested were well detected. It is possible that the drying process and storage may increase the production of autofluorescent compounds. More efforts may be necessary to control this phenomenon.

Of course, we could not evaluate all the markers available for flow cytometry, but we have analyzed more than one hundred markers since we initiated this project and have shown that most applications could be considered. It is likely that other sample types, such as cell lines and bone marrow aspirates, may be amenable to this technique. If preferable from a workflow standpoint, one can also consider staining samples before drying *(data not shown)*.

Using mRNA sequencing, a small leucocyte population such as B cells eluted from the DBS was interrogated for a series of specific gene transcripts. A slight decrease in mRNA quantities was observed in DBS compared to the fresh control, suggesting that the method could be further optimized, for example, by adding different RNase inhibitors.

Using the optimized method, we showed the preservation of the CD169 marker ability to discriminate between patients with and without SARS‐COV‐2. We may consider this method in a context of an active pandemic and lockdown since it would allow self‐sampling/home‐ sampling and shipment to central laboratories. In line with this possibility, we found that blood drawn from a lancet fingerstick and directly spotted onto the solid support can yield cell recovery similar to anticoagulated blood without the need of EDTA pretreatment. Still, a formal evaluation of shipment conditions on the quality of DBS should be performed.

Overall, this study demonstrates that most leucocytes in a drop of blood can be easily stored and shipped using the DBS method. Storage of DBS requires little space, does not necessitate low temperatures, and DBS specimens are considered nonregulated, exempt materials by transportation authorities.^[^ ^27^ ^]^ Finger‐ or heel‐prick sampling is a noninvasive method that gives access to specialized testing to isolated people, patients from remote areas or even the general population requiring some type of screening. Cells recovered in the laboratory can be interrogated for their protein and nucleic acid contents, for a variety of applications encompassing immunological disorders, hematological malignancies and infectious diseases. Ultimately, DBS expands a universal sample storage method for metabolites, proteins, nucleic acids, to include cells.

## 4 Experimental Section

### Blood Samples

For method optimization, blood samples were obtained from healthy volunteers from Saint Joseph Hospital (Marseille, France, declaration DC‐2020‐3857).

Control blood samples were obtained from the blood bank Etablissement Français du Sang (EFS), Marseille, France, agreement No. 7626NQ). Residual volumes of COVID‐19 patient blood samples were obtained from La Timone Hospital – Emergency Department (Marseille, France). The research was approved by the national ethic committee (Agreement ID Recherche Clinique Biomedicale (RCB) No. 2018‐A02706‐49). The studied population included patients older than 18 years with SARS‐CoV‐2‐positive RT‐PCR.

Fingerstick samples and their corresponding venous blood samples were obtained from volunteers at the Beckman Coulter Miami blood sampling center (Kendall, FL).

All enrolled patients provided informed consent and procedures followed were in accordance with the Helsinki Declaration. Care of the subjects was not modified and the results of the study had no influence on subjects’ management. Cell analyses were performed on pseudonymized residual blood and all data collected in the study were retrieved from subject records by the practitioner.

### Recovering Leucocytes from a Dried Blood Spot: Preliminary Testing

Eight different methods have been compared on 6 donors: 20 µL of EDTA blood (Methods 1, 2, 5, and 6) or heparinized blood (Methods 3, 4, 7, and 8) were placed on two types of solid support: cellulose matrix (Whatman filter) from Whatman (Maidstone, UK) (Methods 2, 4, 6, and 8) or polyester matrix (Leukosorb B filter) from Pall (New York, USA) (Methods 1, 3, 5, and 7). Samples were left at least 2 h on the bench for the blood to dry. The solid supports were then stored in nonsterile plastic pouches at RT (18–25 °C) for at least 24 h. To recover the leucocytes, each blood spot (1 cm²) was cut out and placed in a flow cytometry tube (12 × 75 mm, 5 mL). The dried blood was then lysed and stained according to a newly described one‐step staining procedure.^[^ ^28^ ^]^ Briefly, a mix of 1 mL RBC lysing solution (VersaLyse, Beckman Coulter, Brea, CA, USA) with 0,05% FA (Sigma‐Aldrich, St. Louis, USA) and 5 µL of antibodies targeting the pan‐leucocyte marker CD45‐ phycoerythrin (PE)‐cyanin‐7 (PC7), 20 µL of CD15‐fluorescein isothiocyanate (FITC) antibodies targeting neutrophils and eosinophils and 10 µL of CD14‐ phycoerythrin‐Texas Red (ECD) antibodies targeting monocytes, all from Beckman Coulter, were added to the tubes containing the spots. The samples were incubated for 30 min on the bench to enable red blood cells lysis, leucocyte rehydration and staining. The tubes containing the sample were vortexed 5 s X 5 times (Methods 5–8). Alternatively, the tube was shaken manually in an up‐and‐down motion until the solid support disintegrated into small pieces (approximately 15 shakes) (Methods 1–4). The eluates were filtered through CellTrics 50 µm filters from Sysmex (Kobe, Japan) before analysis in a Navios flow cytometer (Beckman Coulter). Leucocytes were gated using Forward Scatter (FSC) and Side Scatter (SSC) parameters, and CD45 expression. Recovery was calculated as the number of leucocytes eluted from the DBS, divided by the number of fresh blood leucocytes counted in the same conditions (20 µL of blood diluted in 1 mL elution buffer).

In order to determine the minimal blood drying timeframe, a few drops of 20 µL blood (allowing a better weighing precision) were spotted on a polyester solid support that was weighed every 30 min.

In order to evaluate cell structure during the drying process, leucocytes were stained every 30 min after spotting, in 1 mL lysing solution 0,05% FA and 5 µL CD45‐ allophycocyanin (APC), and eluted by manual shaking.

Cell permeability was assessed using the nonpermeant DNA stain propidium iodide (PI, Sigma‐Aldrich) at 10 µg mL^−1^.

All experiments were run at RT and local humidity (30–60%). Temperature and humidity levels are routinely recorded in the laboratory using data logger Ecolog TH1 from Elpro (Buchs, Switzerland).

### Method Optimization—Optimal Elution Conditions

Different elution buffers were tested in triplicates: RBC lysing solution, PBS 1X, and PBS 1X supplemented with FA at different concentrations; 0.025%, 0.05%, 0.1%.

Different elution methods were tested in triplicates: vortex (from Bioblock Scientific Heidolph), rotating mixer (from Ratek), automated orbital shaker (Biomek from Beckman Coulter) and manual shaking.

*Method Optimization—Optimal Drying Conditions*: Different solid support pretreatments were tested in triplicates: 20 µL PBS 1X, 20 µL EDTA sodium salt at 1.5–12 mg mL^−1^ from Sigma‐Aldrich, 20 µL lithium heparin at 170 IU from Becton Dickinson (New Jersey, USA), 20 µL of poly‐l‐lysine at 0.1 mg mL^−1^ (Sigma‐Aldrich), and 5, 10, 20, and 40 µL of protease inhibitors 5X (Sigma‐Aldrich) were placed on the solid supports and allowed to dry for at least 2 h before blood spotting.

*Method Optimization—Optimal Storage Conditions*: To determine optimal storage conditions, the DBS were stored in triplicates 7 d in a plastic bag at 4 °C, RT or 37 °C. After storage, leucocytes were recovered, and frequencies and structure measured.

### Cell Marker Preservation Analysis

Fresh and dried blood samples from ten donors were processed in parallel. Fresh EDTA‐anticoagulated blood samples were processed according to the one‐step staining procedure: 20 µL of blood were incubated 15 min in a mix of 1 mL of lysing solution and conjugated antibodies. DBS have been prepared for each fresh EDTA‐ anticoagulated blood sample. The DBS were stored 1 month at RT then processed according to the optimized DBS method: each DBS was cut out and placed in a flow cytometry tube (12 × 75 mm, 5 mL). A mix of 1 mL PBS 1X, 0,05% FA and conjugated antibodies was added to the tube. The samples were then left for 30 min on the bench to enable leucocyte rehydration and staining. The tubes were then shaken up until solid support dissociation then the eluates were filtered through CellTrics 50 µm filters from Sysmex (Kobe, Japan) before analysis in a Navios flow cytometer.

The immune monitoring (IM) Basic Duraclone panel (Beckman Coulter) was used for labeling. The panel contains CD3‐allophycocyanin‐Alexa Fluor‐750 (APC‐A750), CD4‐APC, CD8‐Alexa Fluor‐700 (A700), CD14‐PC7, CD16‐FITC, CD19‐ECD, CD56‐R‐PE, and CD45‐Krome Orange (KrO); all from Beckman Coulter.

Using the same method, intracellular staining was performed after 1 month storage. The following conjugates were used: CD79a‐PE; CD19‐APC, MPO; FoxP3‐Alexa Fluor 647 (A647); CD4‐Pacific Blue; and CD25‐PE; all from Beckman Coulter. For FoxP3 staining, two final washes were added.

### Venous versus Capillary Dried Blood Comparison

EDTA‐anticoagulated venous blood and capillary blood samples have been obtained in parallel from three donors at Miami blood sampling center. Capillary blood samples were spotted in triplicate on three EDTA‐pretreated and three regular polyester solid supports. Venous blood samples were processed fresh, then also dried in triplicate on the regular polyester solid supports. Each solid support was weighed before and right after blood deposit in order to evaluate the volume spotted. All dried samples have then been shipped to Marseille site at RT with an express delivery and analyzed within 5 d. Fresh and dried sample contents were compared with IM Basic Duraclone panel.

### Gene Expression Analysis by Microfluidic Real‐Time PCR

Blood was dried on a polyester solid support under RNase‐free conditions and stored 24 h at RT. Leucocytes were eluted in 1 mL elution buffer (PBS 1 × 0.05% FA) containing 5 µL RNase inhibitors from Applied Biosystems (Foster City, USA) and antibody conjugates CD45‐APC and CD19‐PE all from Beckman Coulter.

Bulk B cell pools of 100 cells were fluorescence‐activated cell sorting (FACS)‐sorted into 96‐well plates (Influx Becton Dickinson cell sorter, Franklin Lakes, USA) containing RT‐preamplification mix and external gene expression primers for selected B cell genes (*CD79B, TCF3, HLA‐DR, CXCR5, HLA‐DOA, IGLL5, SELL*) and housekeeping genes *(GAPDH, B2M)* according to published protocols.^[^ ^29^, ^30^ ^]^ After 20 cycles of cDNA preamplification, the cDNAs were diluted and processed for multiplex gene expression profiling using the BioMark Real‐time PCR system (Fluidigm, San Francisco, USA) using inventoried TaqMan gene expression assay in 48.48 dynamic arrays. Primers and TaqMan assays used for real time quantitative PCR (RT‐qPCR) are displayed in Table S1 in the Supporting Information. The Cycles Threshold (CT) values from each pool were calculated from the BioMark system's software and used as such without normalization.

### CD169 Preservation on DBS from COVID‐19 Patients

Fresh EDTA‐anticoagulated blood samples were processed in parallel according to the one‐step staining procedure and the DBS method.

The results of fresh samples were described,^[^ ^31^ ^]^ and then the DBS were analyzed after one‐week of storage at RT. Both fresh and dried blood were stained with anti‐CD169‐PE and anti‐CD45‐PC7 (Beckman Coulter). CD169 levels are shown as median of fluorescence intensities on monocytes.

### Flow Cytometry Data Analysis and Statistical Analysis

Data were collected on a three‐laser, ten‐color Navios flow cytometer and analyzed using Kaluza Analysis Software (version 2.1; Beckman Coulter).

Comparison of quantitative variables between the different groups was performed on JMP software (version 10; SAS Institute, Cary, NC, USA).

For marker preservation experiments, comparison between leucocyte frequencies on fresh and dried blood were done using Student's *T*‐test; *n* = 10.

The ability of CD169 index to discriminate between COVID‐19+ and COVID‐19‐ patients was investigated using the ROC curve analyses; *n* = 76.

For the elution method optimization and storage temperature experiments, leucocyte recovery percentages were compared using Tukey‐Kramer honestly significant difference (HSD) and subset frequencies were compared using Dunnett's test.; *n* = 3.

For solid support pretreatment experiments, leucocyte recovery percentages were compared on Student's *T‐*test; *n* = 3.

For fingerstick experiments, comparison between subset frequencies in venous and capillary dried blood was done using Student's *T*‐test; *n* = 3.

For all tests, data were presented as mean ± standard deviation. *P*‐values less than or equal to 0.05 were considered statistically significant: *; and *p*‐values higher than 0.05 were considered statistically nonsignificant: ns.

## Conflict of Interest

I.A.B. is recipient of Conventions Industrielles de Formation par la Recherche (CIFRE) Ph.D. grant (No. 2018/1212) from the ANRT (National Agency for Research and Technology). This work was supported by Beckman Coulter through donations of the research reagents used in the flow cytometry experiments and participation of the four employees: I.A.B., P.B., J.M.B., and F.M. The sorting and RT‐qPCR experiments were supported by INSERM and CNRS institutions (S.R., N.M.‐K., and F.G.) and ARC Foundation (S.R.).

## Author Contributions

J.M.B., F.M., I.A.B., S.R., and F.G. designed the study. I.A.B., N.M.‐K., and P.B. conducted experiments and acquired data. P.‐E.M. and P.M. supervised clinical procedures. I.A., M.L., and T.M. collected samples and analyzed demographic and clinical data. I.A.B., P.B., and F.M. provided antibody panels and the flow cytometry platform. N.M.‐K. and S.R. provided the cell sorting and RT qPCR platform. I.A.B., N.M.‐K., P.B., J.M.B., S.R., F.G., and F.M. analyzed experimental data. I.A.B., N.M.‐K., S.R., F.G., and F.M. wrote the paper. Each co‐author has revised the paper for important intellectual content. All authors read and approved the final paper.

## Supporting information

Supporting Information

## Data Availability

The study report, study protocol, and data that support the findings of this study are available in the Supporting Information and from the corresponding author upon reasonable request. The authors certify that this paper reports original research data.
