# Supplementary material for: Cell Analysis from Dried Blood Spots: New Opportunities in Immunology, Hematology, and Infectious Diseases
Source: Adv Sci (Weinh). 2021 Jul 18;8(18):2100323. doi: 10.1002/advs.202100323 (PMC8456206; doi:10.1002/advs.202100323)
Supplement: Supplementary file 1 — Supporting Information [file ADVS-8-2100323-s001.pdf]

© 2021 Wiley-VCH GmbH

ADVANCED SCIENCE
Open Access

Supporting Information

for *Adv. Sci.*, DOI: 10.1002/advs.202100323

Cell analysis from dried blood spots: New opportunities in immunology, hematology and infectious diseases

Ines AIT BELKACEM, Noushine MOSSADEGH-KELLER, Pénélope BOURGOIN, Isabelle ARNOUX, Marie LOOSVELD, Pierre-Emmanuel MORANGE, Thibaut MARKARIAN, Pierre MICHELET, Jean Marc BUSNEL, Sandrine ROULLAND, Franck GALLAND, and Fabrice MALERGUE[*]

SUPPLEMENTARY FIGURES.

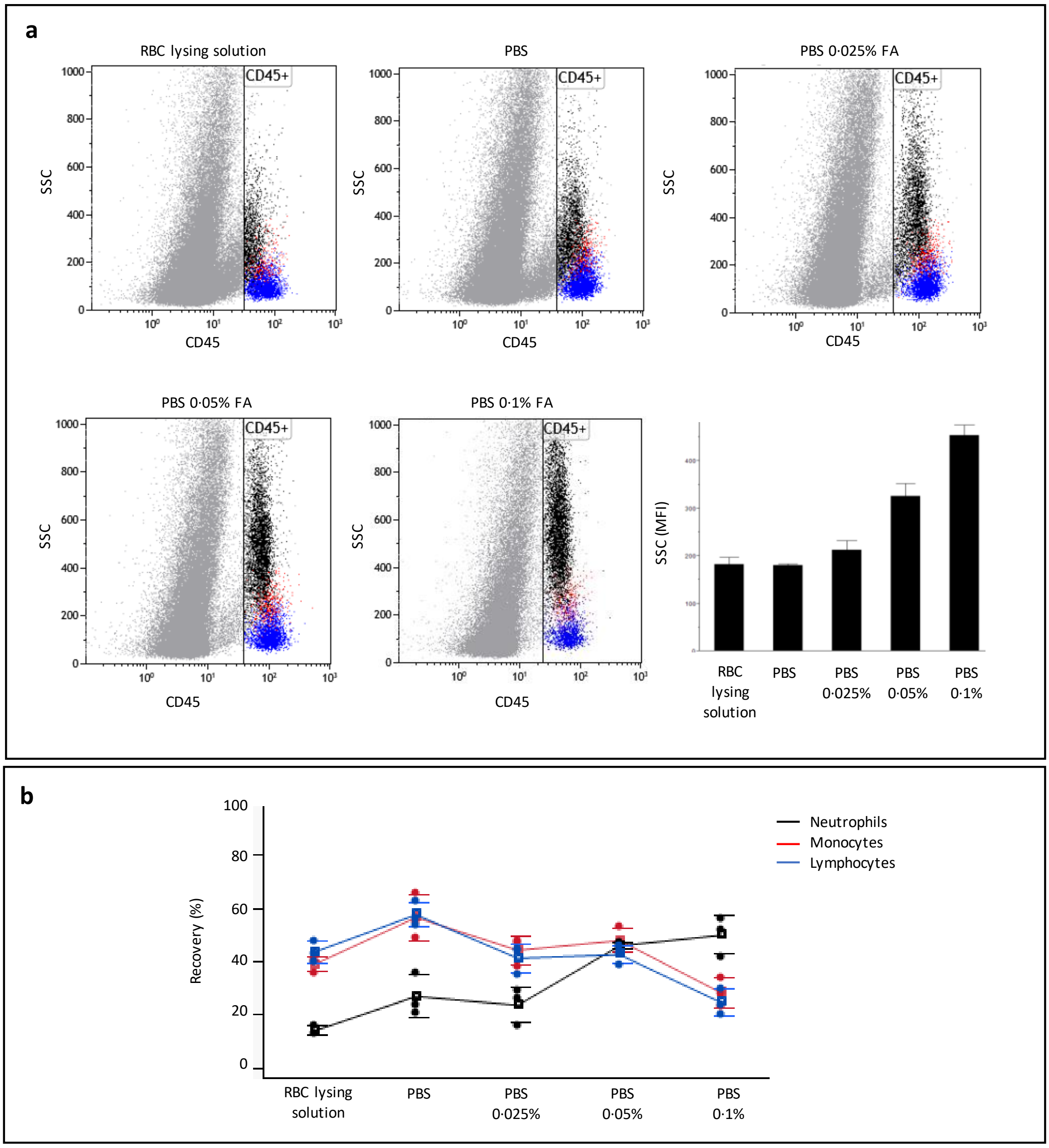

Supplementary Figure 1. Low-concentration formaldehyde improves leucocyte structure. **(a)** Cytometry dot plots and SSC recording on leucocytes eluted from solid support with different elution buffers: RBC lysing solution, PBS 1X, PBS 1X 0,025% FA, PBS 1X 0,05%FA and PBS 1X 0,1%FA, n=3 per condition, and **(b)** corresponding leucocyte subpopulation recovery. All data are presented as mean ± standard deviation.

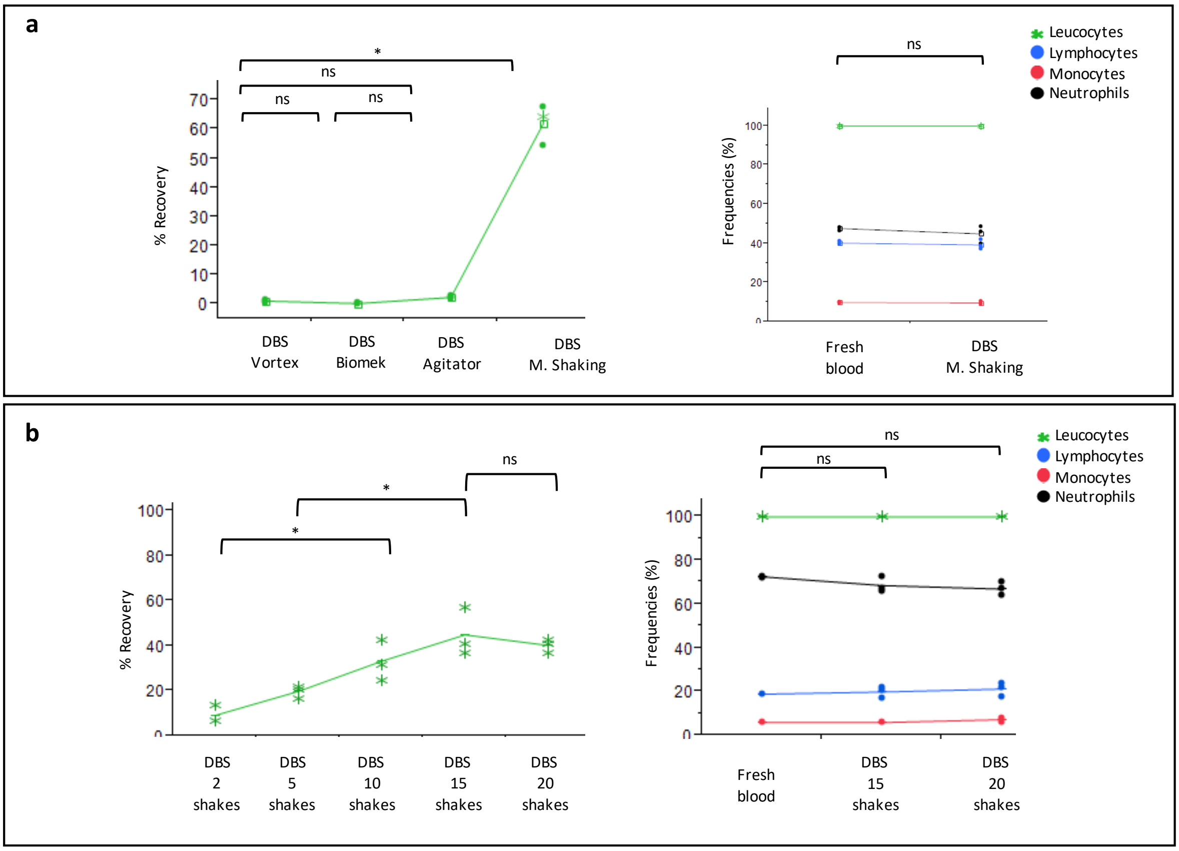

Supplementary Figure 2. Comparing leucocyte recovery with different elution methods**. (a)** Leucocyte recovery and frequencies when eluting cells with vortex, automated orbital shaker, rotating mixer (agitator) and strong manual shaking. **(b)** Leucocyte recovery and frequencies when eluting cells with an increasing number of manual shakes. ns: non-significant (p values > 0,05); *: significant (p values ≤ 0,05), on Tukey-Kramer HSD (for leucocyte recovery) and Dunnett's test (for subset frequencies), n=3 per condition. All data are presented as mean ± standard deviation.

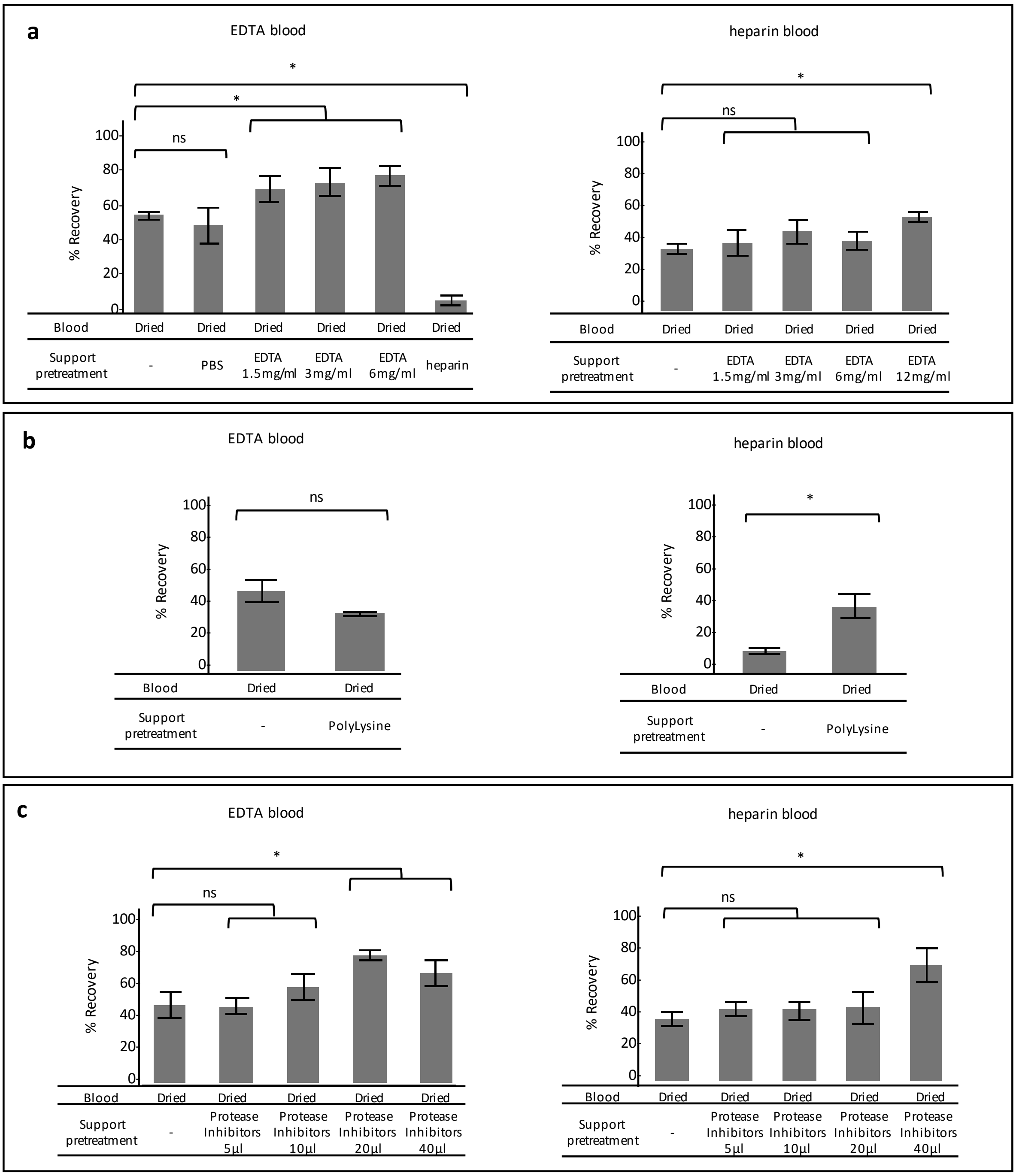

Supplementary Figure 3. Solid support pretreatment effect on leucocyte recovery. Leucocyte recovery in three separate experiments: pretreated solid support with EDTA or heparin **(a)**, poly-L-lysine **(b)** or protease inhibitors **(c).** Mean comparison with control (dried blood without solid support pretreatment) on Student's T test, ns: non-significant (p values > 0,05); *: significant (p values ≤ 0,05), n=3 per condition. All data are presented as mean ± standard deviation.

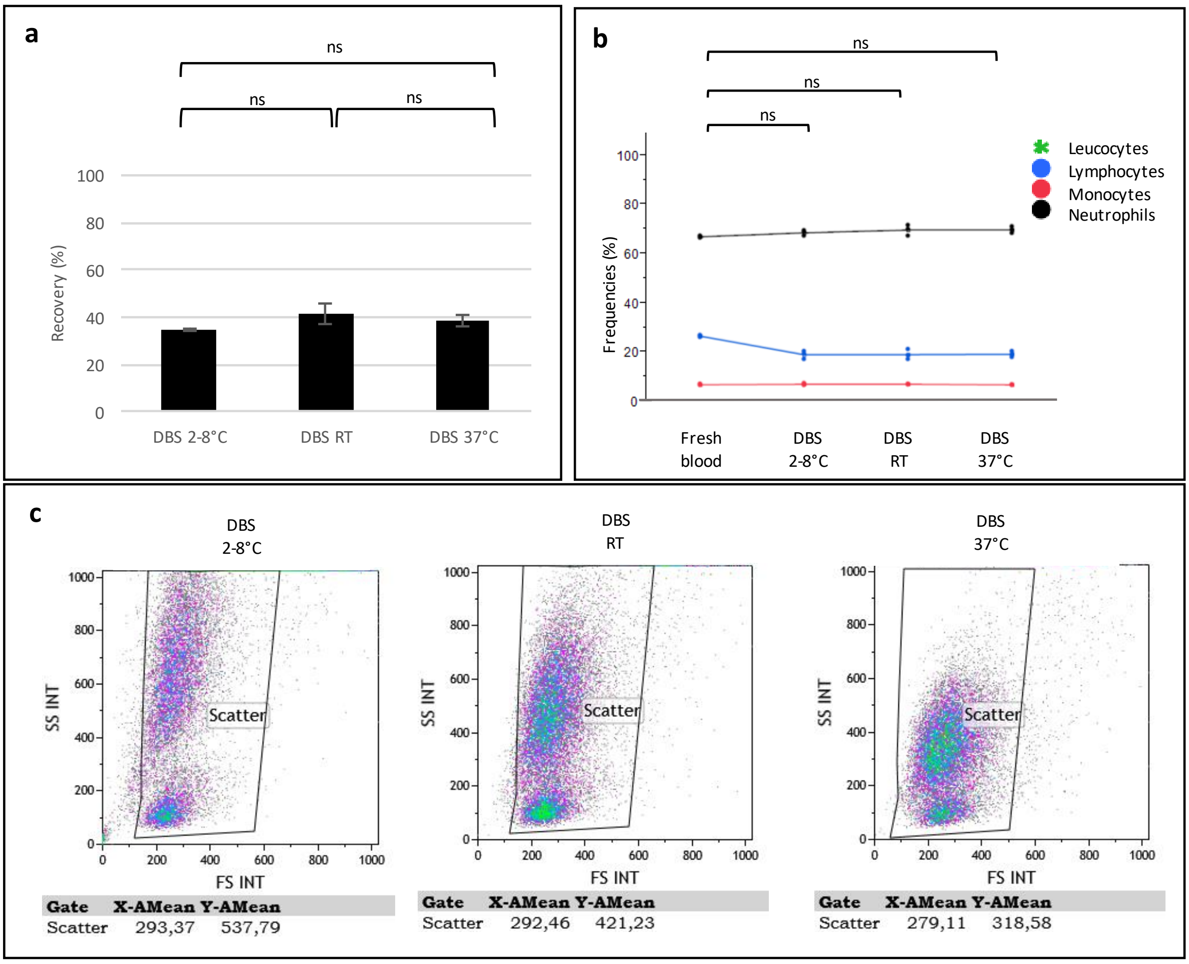

Supplementary Figure 4. Storage temperature effect on leucocyte recovery. **(a)** Leucocyte recovery from DBS stored at 2-8°C, RT and 37°C. Mean comparison with control (RT) on Tukey-Kramer HSD test; ns: non-significant (p values > 0,05); *: significant (p values ≤ 0,05), n=3 per condition **(b)** Leucocyte sub-population proportions on DBS stored at 2-8°C , RT and 37°C. Mean comparison with control (fresh blood) on Dunnett's test; ns: non-significant (p values > 0,05); *: significant (p values ≤ 0,05), n=3 per condition. **(c)** Cytometry dot plots (FSC: SS) on DBS stored at 2-8°C, RT and 37°. All data are presented as mean ± standard deviation.

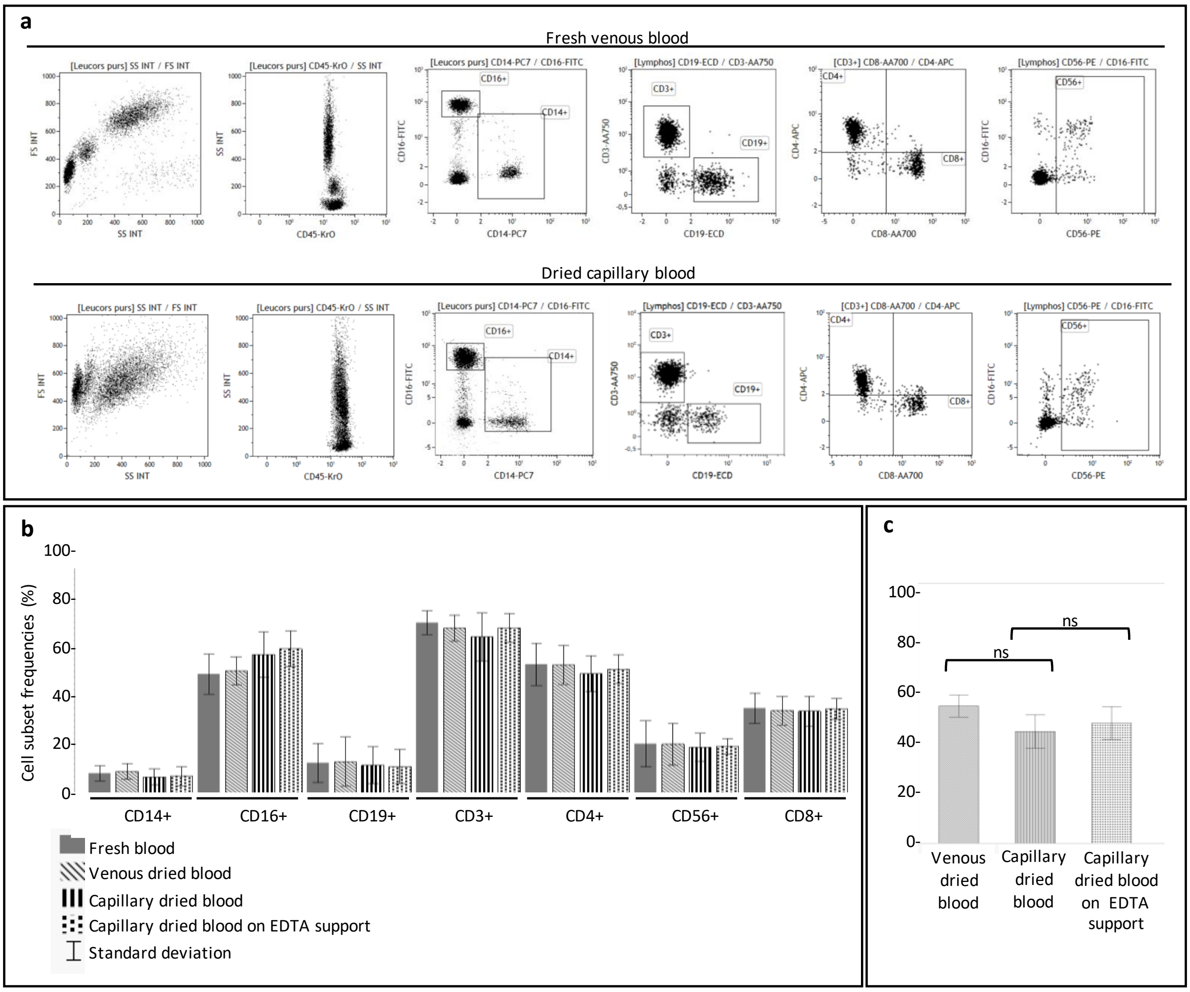

Supplementary Figure 5. Venous versus capillary dried blood comparison. Cell markers **(a)**, corresponding subset frequencies **(b)**, and leucocyte recovery **(c)** from fresh blood, dried venous blood, and fingersticks dried with or without EDTA solid support pretreatment. Comparison between the different conditions on Student’s T test, ns: non-significant (p values > 0,05); *: significant (p values ≤ 0,05), n=3 donors per condition, data are presented as mean ± standard deviation.

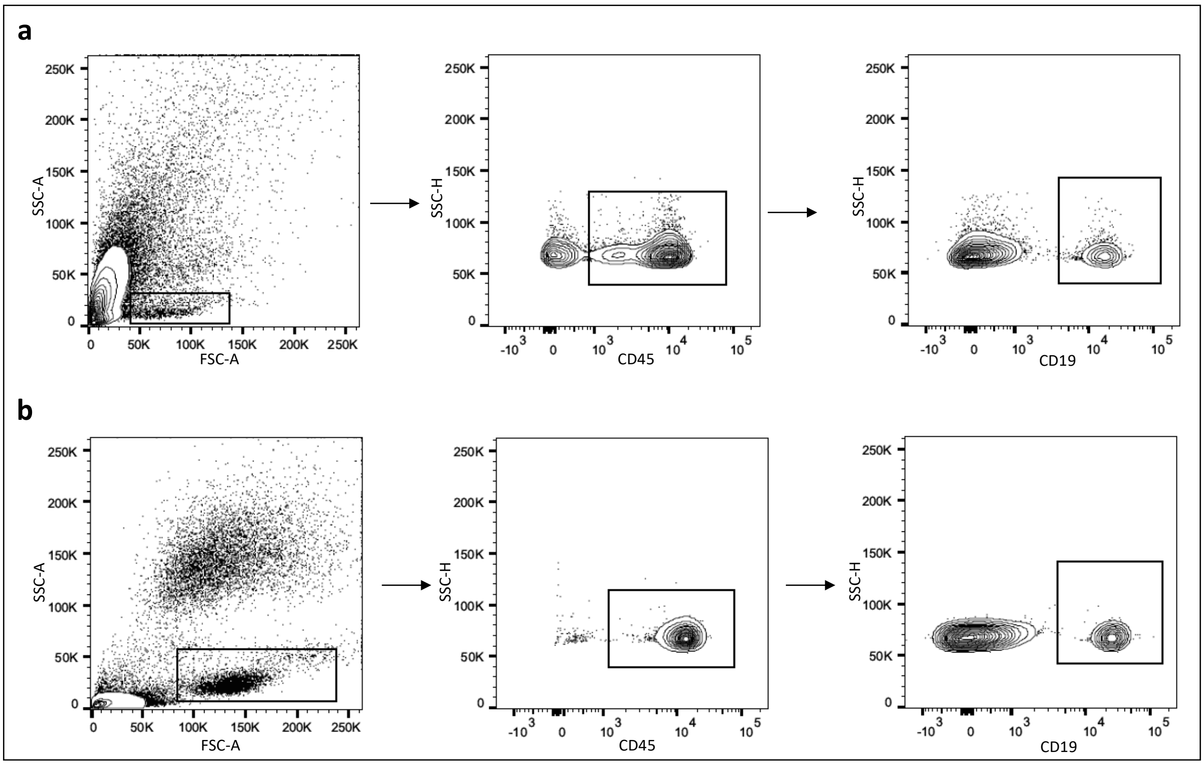

Supplementary Figure 6. Representative FACS profiles of cells collected from DBS **(a)** versus from fresh venous blood **(b)** CD45+CD19+ B cell populations isolated from donor C.

Supplementary Table 1. Subset frequencies in fresh and dried blood

	Donor 1		Donor 2		Donor 3		Donor 4		Donor 5	
	**Fresh**	**Dried**	**Fresh**	**Dried**	**Fresh**	**Dried**	**Fresh**	**Dried**	**Fresh**	**Dried**
CD14+	9,99	7,31	7,36	5	8,14	6,73	6,16	4,41	4,24	3,28
CD16+	47,2	68,28	63,41	80,32	51,35	67,34	66,15	76,49	73,26	80,06
CD19+	9,25	9,24	13,1	12,99	5,76	5	12,8	12,19	11,67	11,05
CD3+	83,79	80,7	69,63	59,53	52,71	47,93	66,03	58,24	72,47	68,79
CD4+	48,98	47,79	60,77	59,97	48	43,92	49,15	50,3	62,78	60,98
CD8+	44,76	43,03	34,14	32,18	45,67	47,78	39,83	38,2	33,88	33,41
CD56+	11,23	11,98	13,43	13,15	40,94	42,27	31,28	29,68	13,36	13,25
	**Donor 6**		**Donor 7**		**Donor 8**		**Donor 9**		**Donor 10**	
	**Fresh**	**Dried**	**Fresh**	**Dried**	**Fresh**	**Dried**	**Fresh**	**Dried**	**Fresh**	**Dried**
CD14+	9,44	3,88	6,35	5,41	5,23	4,07	10,47	8,07	6,1	5,04
CD16+	53,45	80,45	59,29	73,04	77,17	86,37	71,6	79,23	79,03	83,18
CD19+	11,15	12,03	19,99	23,46	7,93	9,96	10,06	10,88	8,26	9,23
CD3+	79,75	74,77	63,19	55,18	83,42	73,8	82,91	79,43	68,38	61,94
CD4+	61,47	60	75,29	73,92	60,73	63,22	62,18	62,32	62,3	60,32
CD8+	28,99	27,03	21,2	17,89	35,39	27,9	31,12	31,11	25,73	25,81
CD56+	17,26	15,89	14,79	17,74	7,58	9,83	15,46	12,86	22,07	21,41

Supplementary Table 2. Gene-specific primers and TaqMan assays

Gene name	External Forward primer (5'->3')	External Reverse primer (5'->3')	TaqMan assay
B2M	ACACTGAATTCACCCCCACT	ACATGGAGACAGCACTCAAAGT	Hs00984230_m1
CD79B	ATCATGATCCAGACGCTGCT	TGGGTGCTCACCTACAGACC	Hs01058826_g1
CXCR5	GATCAATCAAACCCGGCGGT	TTCCCTGCCTCAGTGTGTTTC	Hs00540548_s1
GAPDH	CATGGGTGTGAACCATGAGA	TTCAGCTCAGGGATGACCTT	Hs02758991_g1
HLA-DOA	GCGCCACTCCTCAGGCATT	AAAGTCAGCACAGCGGGATG	Hs01109372_g1
HLA-DRA	CACTCCCGAGCTCTACTGAC	CCAAATTCTTCAAGCCGCCA	Hs00219575_m1
IGLL5	CTGCTGCGCCCAATGGTT	CAGGCCACTGTCACAGCTC	Hs04330879_u1
SELL	TTTGGGCAAGGACCTGAGAC	GGTTCCATGATGTGCCAGGA	Hs00174151_m1
TCF3	GCACTGGCCTCGATCTACTC	CCTCGTCCAGGTGGTCTTCT	Hs00413032_m1
